# Online Search Behavior Related to COVID-19 Vaccines: Infodemiology Study

**DOI:** 10.2196/32127

**Published:** 2021-11-12

**Authors:** Lawrence An, Daniel M Russell, Rada Mihalcea, Elizabeth Bacon, Scott Huffman, Ken Resnicow

**Affiliations:** 1 Center for Health Communications Research Rogel Cancer Center University of Michigan Ann Arbor, MI United States; 2 Division of General Medicine School of Medicine University of Michigan Ann Arbor, MI United States; 3 Google Mountain View, CA United States; 4 Computer Science and Engineering Division College of Engineering University of Michigan Ann Arbor, MI United States; 5 Department of Health Behavior & Health Education University of Michigan School of Public Health Ann Arbor, MI United States

**Keywords:** online health information, behavior, search, COVID-19, vaccine, infodemiology, internet, trend, public health, awareness, concern, interest, public, misinformation, safety, side effect, availability

## Abstract

**Background:**

Vaccination against COVID-19 is an important public health strategy to address the ongoing pandemic. Examination of online search behavior related to COVID-19 vaccines can provide insights into the public's awareness, concerns, and interest regarding COVID-19 vaccination.

**Objective:**

The aim of this study is to describe online search behavior related to COVID-19 vaccines during the start of public vaccination efforts in the United States.

**Methods:**

We examined Google Trends data from January 1, 2021, through March 16, 2021, to determine the relative search volume for vaccine-related searches on the internet. We also examined search query log data for COVID-19 vaccine-related searches and identified 5 categories of searches: (1) general or other information, (2) vaccine availability, (3) vaccine manufacturer, (4) vaccine side-effects and safety, and (5) vaccine myths and conspiracy beliefs. In this paper, we report on the proportion and trends for these different categories of vaccine-related searches.

**Results:**

In the first quarter of 2021, the proportion of all web-based search queries related to COVID-19 vaccines increased from approximately 10% to nearly 50% of all COVID-19–related queries (*P*<.001). A majority of COVID-19 vaccine queries addressed vaccine availability, and there was a particularly notable increase in the proportion of queries that included the name of a specific pharmacy (from 6% to 27%; *P*=.01). Queries related to vaccine safety and side-effects (<5% of total queries) or specific vaccine-related myths (<1% of total queries) were uncommon, and the relative frequency of both types of searches decreased during the study period.

**Conclusions:**

This study demonstrates an increase in online search behavior related to COVID-19 vaccination in early 2021 along with an increase in the proportion of searches related to vaccine availability at pharmacies. These findings are consistent with an increase in public interest and intention to get vaccinated during the initial phase of public COVID-19 vaccination efforts.

## Introduction

We are currently in the midst of a global pandemic caused by COVID-19. At all times, and particularly during a pandemic, it is critical for the public to have access to timely and accurate health information [1-3]. The internet is a major source of such health information [4-7]. Analysis of health-related web search behavior can provide critical insight into the public’s awareness and interest in specific health issues and their health concerns and information needs, health experiences, and health-related intentions and behaviors [8-11].

Infodemiology is the scientific study of the “distribution and determinants of information in an electronic medium, specifically the internet, or in a population, with the ultimate aim to inform public health and health policy” [8]. To date, a number of infodemiology studies related to the COVID-19 pandemic have been published. Many of these studies identified an association between general COVID-19–related or symptom-specific search trends and COVID-19 case incidence and associated deaths [12-18]. In several cases, search behavior appeared to be an effective predictor of disease trends. Several studies have examined the occurrence and spread of COVID-19–related misinformation, which has been a public health challenge during the pandemic [19-25]. Another group of studies has examined search behavior to gain insights into public awareness, interest, attitudes, and behaviors related to COVID-19 [21,26-28]. Husain et al [27] found that countries that demonstrated a more rapid increase in public search interest regarding COVID-19 also tended to be more effective in their control of the pandemic.

Vaccination against COVID-19 is a major public health strategy in the effort to end the pandemic [29]. As of Spring 2021, a number of effective COVID-19 vaccines have been available that substantially reduce the risk of COVID-19–related illness, hospitalizations, and death [30]. Understanding public awareness and interest in COVID-19 vaccines and willingness to vaccinate are critical to help guide vaccination efforts. Unfortunately, vaccine hesitancy is common and poses a major barrier to successful vaccination efforts [31]. Infodemiologic approaches can provide potentially important insights into the public’s awareness, interests, concerns, and intentions related to COVID-19 vaccination. Thus far, few infodemiological studies have focused on COVID-19 vaccines [32,33]. To address this critical gap, we examined the relative search volume using Google Trends data and also search query logs capturing users’ online search behaviors related to COVID-19 vaccines in the first quarter of 2021.

## Methods

### Study Design

This study describes users’ online search behavior via Google search engine for searches related to COVID-19 vaccines in the first quarter of 2021. Google is the dominant search engine in the United States, accounting for approximately 89% of the total search volume in the country as of January 2021 [34]. We focused on the time period from January 1, 2021, through March 16, 2021, which follows the initial emergency-use authorization by the US Food and Drug Administration (FDA) for the Pfizer and Moderna COVID-19 vaccines and the start of public vaccination efforts.

### Data Sources

#### Google Trends

Google Trends provides open access to time-series data related to Google search engine search volumes for specific terms [35]. Search query volume was normalized to a percentage scale (0% to 100%) to provide a measure of relative search volume (RSV), with 100% corresponding to the peak in search volume in any given time frame for that specific topic. By searching for multiple terms simultaneously, we were able to compare the RSV for different terms. For this report, we focus on Google Trends data for COVID-19 vaccine-related searches in the United States.

#### Search Query Logs

**Figure 1:**
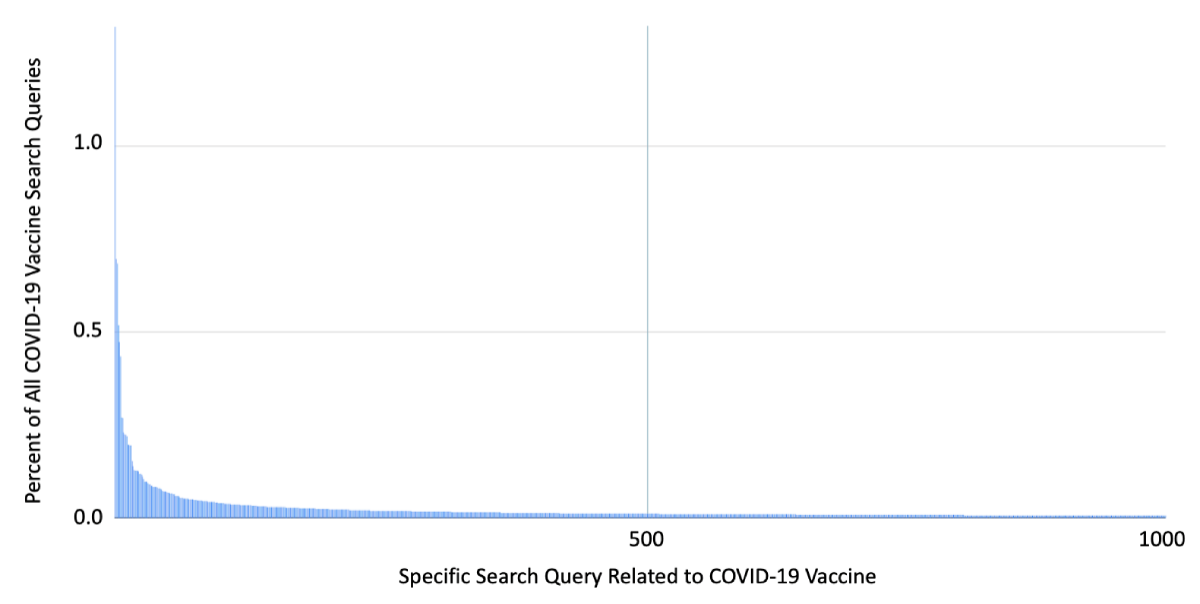
Frequency distribution of the 1000 most common search queries related to "COVID-19 vaccine.".

Search engine query logs record the specific language that users employ when conducting online searches and can provide insight into the users' information needs and interests and how these change over time [36-38]. To examine COVID-19 vaccine-related search behavior in greater detail, we compiled anonymized data from Google search query logs. That is, we examined a complete sample of English-language queries conducted in the United States during the search period (January 1, 2021, to March 16, 2021). We collected only queries that contain both the terms “COVID” and “vaccine.” This data set comprises over 45.4 million queries during the sampling period, which suggests that when people search for information about COVID-19 vaccines, they use a fairly limited number of common queries. For example, the top 150 most common queries related to COVID-19 account for approximately half of all queries in the data set. The distribution of search queries by volume is shown in Figure 1. This figure shows that a small number of queries accounts for a large proportion of the overall query volume. This query volume distribution curve essentially becomes asymptotic after the top 1000 most common queries. To create this search query log data set, we collected the top 5000 most common COVID-19 vaccine-related search queries for each day during the study period.

### Metrics

#### Overview

Metrics of interest for this study are based upon both Google Trends and the search query log data set. Specific Google Trend metrics include (1) comparison of the RSV for any searches related to COVID-19 and those related to COVID-19 vaccine, (2) general RSV for the term “vaccine” since 2005, and (3) comparison of RSV for specific vaccine myths and conspiracy beliefs identified in the search query logs.

Our main interest with the search query log data set is to examine the distribution and trends of different types of COVID-19 vaccine-related searches. We developed a COVID-19 vaccine search query classifier using the following steps.

#### Step 1: Identify Search Categories

Two study authors (DMR and LA) performed independent manual review of a random sample of 1000 queries to identify common themes, as well as unique terms associated with each category. The two authors met to review and resolve any discrepancies and reached complete agreement on both categories and associated terms. Based on this review, we identified the following categories of search queries: (1) vaccine availability, (2) vaccine maker or manufacturer, (3) vaccine side effects or safety, (4) vaccine myth or conspiracy beliefs, and (5) general or other vaccine-related searches. A definition of each of these categories of searches, associated search terms, and examples is shown in Table 1. Because pharmacies were a major channel for distribution of COVID-19 vaccines, we also created a subcategory of vaccine availability queries that asked about COVID-19 vaccines in relation to pharmacies (eg, included the name of specific pharmacy chains).

**Table 1:** Types of COVID-19 vaccine–related search queries.

Category	Definition	Associated terms	Examples of specific queries
Availability	Query that included a term or phrase identifying locations where or time when COVID-19 vaccines might be available	Names of US states, counties, or cities, names of organizations or specific locations that provide COVID-19 vaccines (eg, pharmacies, hospitals or health systems, vaccination sites), when or where to get COVID-19 vaccines	“ny covid vaccine”, “covid vaccine california”, “florida covid vaccine”, “covid vaccine near me”, “where to get covid vaccine”, “cvs covid vaccine”, “covid vaccine rite-aid”, “covid vaccine appointment”, “when can I get covid vaccine”
Maker or manufacturer	Query that included the name of a COVID-19 vaccine maker or manufacturer	Names of different COVID-19 vaccines, names of companies or organizations that developed or manufactured different vaccines	“pfizer vaccine”, “moderna vaccine”, “johnson vaccine”, “j&j vaccine”
Side effects or safety	Query that included general or specific terms associated with side effects or safety of COVID-19 vaccines	Side effects, safety, specific vaccine-related worries and concerns	“covid vaccine side effects”, “covid vaccine safety”, “reaction to covid vaccine”, “pregnant women covid vaccine”, “covid vaccine blood clot”, “problems with covid vaccine”, “covid vaccine fever”, “covid vaccine allergy”
Myths or conspiracies	Query that included general or specific terms associated with COVID-19 vaccine myths or conspiracy beliefs	Specific myths or conspiracy beliefs	“covid vaccine infertility”, “does covid vaccine change dna”, “covid vaccine microchip”, “can I get covid from vaccine”, “covid vaccine 5G”
General or other	Query related to COVID-19 vaccine that included no additional terms or terms not associated with any of the above categories	COVID-19 vaccine or vaccination or other topics other than identified above	“covid vaccine”, “covid-19 vaccine”, “coronavirus vaccine”, “covid vaccine update”, “covid vaccination rates”

#### Step 2: Identify Terms Associated With Each Search Category

One study author (LA) then manually reviewed an additional random sample of 5000 queries to identify any additional terms that might be associated with each search category. The results of this review were discussed with additional authors (DMR, KR, and RM) to create a final list of unique search query terms associated with each search category.

#### Step 3: Create and Apply Search Query Classifier

We created a rules-based classifier that assigned a query to one or more of the 5 categories based on the presence of a unique set of associated terms while accounting for common variations in spelling. Some search queries contained terms associated with multiple categories, and these queries were counted separately in each appropriate category. For example, a search for “Pfizer covid vaccine CVS” would be counted as a query related to vaccine availability (given the presence of the name of a specific pharmacy) and also as a query related to vaccine manufacturer H (given the presence of the name of a specific vaccine manufacturer).

#### Step 4: Evaluate Performance of the Search Query Classifier

This classifier was able to classify 90% of all queries in the entire COVID-19 vaccine search query log dataset. The remaining 10% of unclassified queries represented searches for additional vaccine-related information (eg, “covid vaccination rates,” or “how long does the covid vaccine last”) and are labeled as “other” and included as part of the category of “general or other” searches. After application of the classifier, an additional random sample of 1000 search queries with classifier results was reviewed separately by 2 authors (DMR and LA) to assess the accuracy of the classifier. These authors met to resolve any discrepancies in manual review and the results of this review were used to calculate the classifier's precision and recall for each of the search categories. The classifier performed well with precision of 99.8% to 100% and recall over 99.5% across all search query categories.

### Analysis

Based on the search query log data set, we employed linear regression to examine the time trends for the proportion of different types of COVID-19 vaccine-related searches over time. For each week during the study period, we calculated a proportion corresponding to each of our categories of interest. The proportions examined in these analyses correspond to the above-described categories. The proportion of COVID-19 vaccine-related searches in each of these categories serves as the dependent variable in separate linear regression models. In each of these models, time (ie, week number since start of the study period) serves as the independent variable to examine the significance of trends over time.

### Ethics Review

This was reviewed by the University of Michigan Institutional Review Board and judged to be exempt based upon its use of open access and anonymized aggregate data.

## Results

### Overview

**Figure 2:**
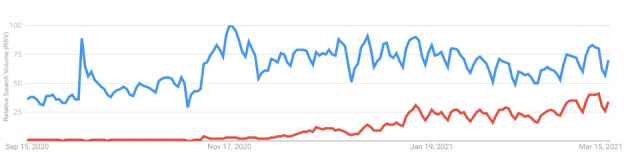
Relative search volume (RSV) for the terms “COVID” (blue) and “COVID vaccine” (red) from September 2020 through March 2021.

The relative search volumes for any searches related to COVID-19 and those related to COVID-19 vaccines are shown in Figure 2. This figure shows a clear increase in the relative volume of COVID-19 vaccine–related searches over the study period.

In the beginning of January 2021, approximately 10% of all COVID-19–related queries were about vaccines. By March 2021, nearly 50% of all COVID-19–related searches were vaccine related. A linear regression was calculated to predict the fraction of queries about COVID-19 vaccines based on daily change during the sample period. A significant linear regression model was found (df=103; *R^2^*=0.76; beta coefficient for time=.31; *P*<.001), indicating that the RSV for COVID-19 vaccine queries increased over the study period.

**Figure 3:**
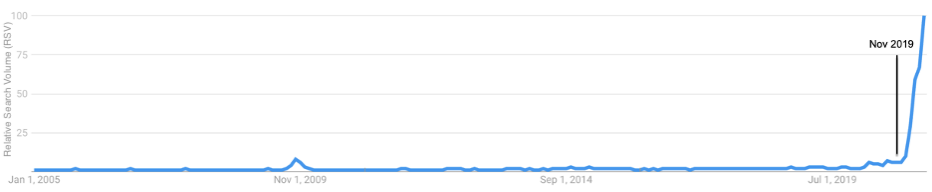
Relative search volume for the term “vaccine” from January 2005 through March 2021.

Figure 3 provides a broader historical context for the level of vaccine-related search interest. This figure shows the relative search volume for the term “vaccine” from January 2005 through the first quarter of 2021. The small peak in vaccine-related search volume occurring in October 2009 coincides with the H1N1 influenza epidemic [39]. The peak in vaccine-related searches in early 2021 is several fold higher than this prior peak in 2009.

**Figure 4:**
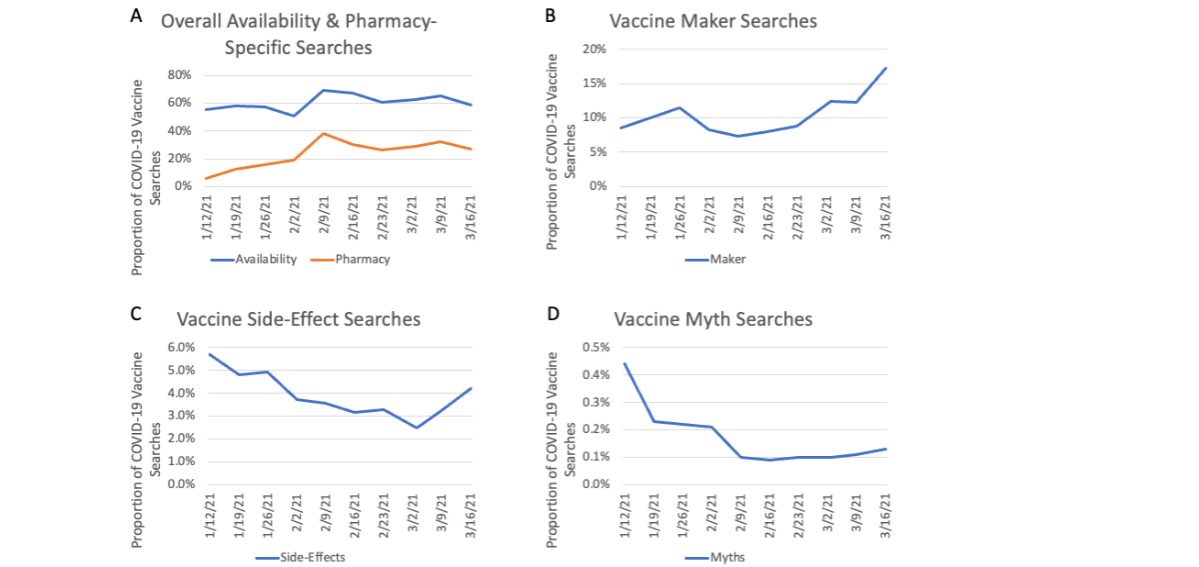
Trends for different categories of COVID-19 vaccine search queries: (A) overall availability and pharmacy, (B) vaccine manufacturer, (C) side effects and safety, and (D) myths and conspiracy beliefs.

A breakdown of the proportion and trends for different types of COVID-19 vaccine-related queries based upon search query log data is shown in Figure 4. Trends for the proportions of different categories of COVID-19 vaccine-related searches are described below.

### Vaccine Availability

During the study period, a majority of searches were classified as related to vaccine availability, with the specific proportion ranging from 55% to 69% each week (Figure 4A). The high proportion of vaccine availability searches was consistent over the study period. Linear regression showed that the time trend for the proportion of queries related to vaccine availability during the study period was not significant (df=8; *R^2^*=0.19; beta coefficient for time=.81; *P*=.20).

During the study period, there was a substantial increase in the subcategory of searches related to pharmacies. The proportion of COVID-19 vaccine-related queries that included a specific pharmacy name increased from 5.9% at the start of the study period to 27.2% at the end of the study period (Figure 4A). Linear regression showed the time trend for this change was positive and significant (df=8; *R^2^*=0.56; beta coefficient for time=2.51; *P*=.01).

### Vaccine Manufacturers

Over the same period, the proportion of vaccine manufacturer-related searches (eg, Pfizer, Moderna, Janssen or Johnson & Johnson) averaged 10.4%. Linear regression shows that the time trend for the proportion of vaccine manufacturer-related searches during the study period was not significant (df=8; *R^2^*=0.38; beta coefficient for time=.61; *P*=.06).

### Side Effects and Safety

The proportion of searches related to side effects or safety of COVID-19 vaccines was quite small, and the proportion actually decreased slightly (from 5.7% to 4.2%) over the study period. Linear regression showed a negative time trend for the proportion of queries related to vaccine side effects or safety during the study period (df=8; *R^2^*=0.51; beta coefficient for time=–0.23; *P*=.02).

### Myths

**Figure 5:**
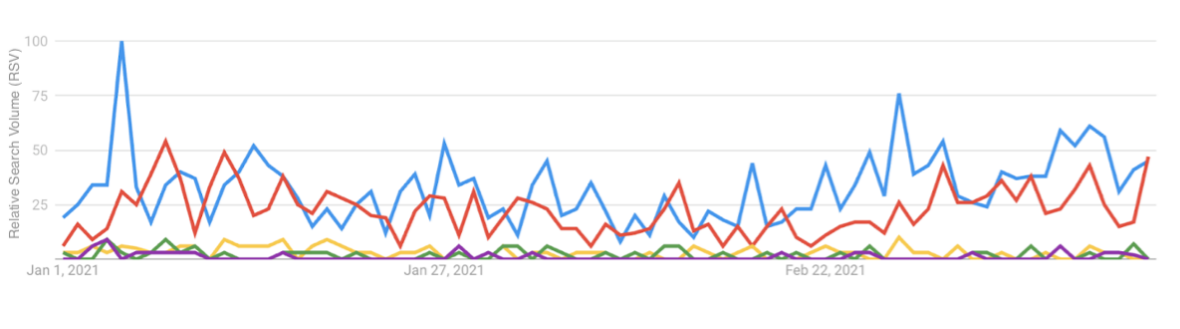
Google Trends relative search volume for queries related to specific COVID-19 vaccine myths and conspiracy beliefs (red: vaccine + DNA, blue: vaccine + fertility, green: vaccine + microchip, gold: vaccine + 5G, and purple: “COVID from vaccine”).

During the study period, the overall proportion of COVID-19 vaccine-related queries that included mention of myths or conspiracies related to COVID-19 vaccines was quite low. This proportion actually decreased slightly (from 0.4% to 0.1%) over the study period. Linear regression showed a negative time trend for the proportion of queries related to vaccine myths or conspiracy beliefs during the study period (df=8; *R^2^*=0.58; beta coefficient for time=–0.01; *P*=.01). Searches related to specific myths or conspiracy beliefs included searches related to the COVID-19 vaccine and (1) infertility, (2) potential to cause change in DNA, (3) 5G and the vaccine, (4) microchips, and (5) contracting COVID-19 from the vaccine itself. The Google Trends RSV for searches related to these specific myths and conspiracy topics is presented in Figure 5. This figure reveals that searches related to COVID-19 vaccine and “infertility” and “DNA” were the most common vaccine myth-related searches.

## Discussion

### Principal Findings

This study reports on online search behavior related to COVID-19 vaccines in the United States, from the first quarter of 2021. During this period, there was a clear increase in the volume of online searches for information about COVID-19 vaccines with a consistently high proportion of searches related to vaccine availability. Online search behavior is influenced strongly by external events and associated media coverage [40,41]. Critical events that are likely drivers of the observed patterns in online search behavior include emergency-use authorizations by the US FDA for 3 different vaccines (ie, Pfizer on December 11, 2020, Moderna on December 18, 2020, Johnson & Johnson on February 26, 2021) and the beginning of public vaccination efforts. During this time, web-based registration was also one of the major means to obtain a vaccine appointment. A particularly notable rise in the proportion of COVID-19 vaccine searches that include the names of specific pharmacies is consistent with the US national strategy that featured pharmacies as vaccination sites.

We interpret these patterns of online search behavior related to COVID-19 vaccines, particularly the rise in pharmacy-related searches, as a sign of increased readiness and intentions to vaccinate among the US population during the study period. This interpretation is consistent with the findings of national tracking surveys in the United States that demonstrate a similar increase in intentions to vaccinate over the study period. Specifically, the Kaiser Family Foundation COVID-19 Tracking Survey shows that the proportion of US adults that either had been vaccinated or would want to get vaccinated as soon as possible increased from under 40% to over 60% during the study period [42]. The increase in online searches related to COVID-19 vaccines is also consistent with national survey findings that reported an increase in the proportion of US adults who reported they had “enough information about when or where to get the vaccine” also over the same period [42].

It is interesting to consider how to interpret our findings regarding online search behavior related to information about COVID-19 vaccine side effects and safety. National surveys conducted during the study period show that among the majority of adults who had not yet been vaccinated, a substantial proportion were concerned about the long-term effects of COVID-19 vaccines (68%), the potential for serious side effects from COVID-19 (59%), or that the vaccines may not be safe (55%) [43]. Given the prevalence of these concerns about COVID-19 vaccine side effects and safety, the relatively low proportion and decreasing time trend for vaccine-related searches that addressed these topics is somewhat surprising. The low proportion of COVID-19 vaccine-related searches pertaining to side effects or safety could be due to a relatively low rate of active information seeking about these aspects among those who are hesitant to get the vaccine, active searching on the web for vaccine appointments among those individuals highly motivated to obtain the vaccine, or some combination of these factors. In such a situation (where some segments of the population are highly motivated to search actively for information while others are not), the relative frequency of searches related to different topics does not appear to provide a good representation of the level of public concern or interest in these topics.

Our findings regarding the frequency of online searches related to COVID-19 vaccine myths or conspiracy theories can be similarly interpreted. Belief, or at least uncertainty, regarding COVID-19 vaccine myths is unfortunately common. During the same time period as this study, national surveys show that 34% of adults in the United States who had not been vaccinated either believed or were unsure about one or more common COVID-19 vaccine-related myths [43]. Nevertheless, at the same time, we found less than 1% of online searches related to COVID-19 vaccines addressed these topics and that this proportion actually decreased over the study period. These findings suggest that many individuals who either believe or are unsure about COVID-19 vaccine myths are not actively seeking additional information online to help clarify their understanding. Although making inferences regarding the population prevalence of a particular COVID-19 vaccine myth may be difficult based upon analysis of search behavior, it is possible that relative search volume might be useful in helping to assess which particular myths are more or less common. For example, the results of our RSV comparison for specific vaccine myths, direct evaluation of COVID-19 vaccine misinformation on the web, and national population surveys identify concerns about infertility related to COVID-19 vaccines as among the most common [33,43].

### Limitations

Several limitations need to be considered while interpreting the results of this study. First, it is important to acknowledge these findings are based on an analysis of search behavior in the United States and are likely to be influenced by specific vaccine approvals and distribution plans in this country. Future work is needed to determine how COVID-19–related vaccine search behavior might differ across countries. Second, these results apply only to search queries conducted using the Google search engine. Although Google is the dominant search engine in the United States, future work is needed to understand how search behavior described here is similar or different for other search engines or on other platforms. For example, social media has been identified as a major source of exposure to COVID-19–related misinformation. Our finding of relatively low rates of active searching for COVID-19 vaccine myths and conspiracy beliefs might or might not apply to search behavior within social media platforms. Third, it is important to acknowledge the subjective nature of our approach for identifying and defining search themes and categories. Other teams could have certainly chosen to identify or define search categories (or subcategories) in other ways. Fourth, of relevance, we also acknowledge the uncertainty regarding our interpretation of the observed patterns of online search behavior representing an increase in interest or intention among the study population to take the COVID-19 vaccine. Although we believe the major study finding that a large increase specifically in pharmacy-related COVID-19 vaccine queries strongly suggests active searching on the web for the vaccine, future work that directly assesses users' information needs (eg, near-time or real-time surveys) would be needed to confirm this interpretation. Finally, it is important to note that the results presented here are based on the aggregate search volume measured at the population level. We, therefore, are not able to determine the degree to which changing patterns in online search behavior are due to changes in the number of individuals performing a specific search, or due to an increase in the number of searches performed by specific individuals, or some combination of these factors.

### Conclusions

Despite these limitations, the findings presented here provide important information about the use of an infodemiologic approach to assess COVID-19 vaccine-related interest and intentions. During the study period, online search behavior related to COVID-19 vaccines suggested a possible historic high in public interest of vaccines. Furthermore, the specific type of vaccine related searches (eg, increased searches related to specific pharmacies and decreased searches related to vaccine side effects) is consistent with reduced vaccine hesitancy and greater intention to vaccinate. The relatively low occurrence of some types of searches (eg, COVID-19 vaccine myths and conspiracy beliefs) suggests that many individuals who lack or are uncertain about critical vaccine-related information are not engaged in active online search to address their information needs. Encouraging more active information-seeking, along with critical appraisal of health information on the web, could be an important strategy to combat misinformation about COVID-19 vaccines and increase vaccine confidence and intention to vaccinate among the general population.

## Abbreviations

FDA: Food and Drug Administration
RSV: relative search volume
